# The Influence of Dry Hydrated Limes on the Fresh and Hardened Properties of Architectural Injection Grout

**DOI:** 10.3390/ma14195585

**Published:** 2021-09-26

**Authors:** Andreja Padovnik, Violeta Bokan-Bosiljkov

**Affiliations:** Faculty of Civil and Geodetic Engineering, University of Ljubljana, Jamova 2, SI-1000 Ljubljana, Slovenia; violeta.bokan-bosiljkov@fgg.uni-lj.si

**Keywords:** architectural injection grout, dry hydrated lime, particle density, specific surface area, workability, porosity, mechanical properties

## Abstract

Dry hydrated lime is an air binder often used in architectural injection grouts. This study compared the influences of three commercially available dry hydrated limes on the injection grouts’ workability and mechanical properties. The main differences between the limes were in their chemical and mineralogical composition and Blaine specific surface area. The grouts were composed of dry hydrated lime, finely ground limestone filler, water, and super plasticiser. Subsequent results obtained revealed that the Blaine specific surface area is not directly related to the fresh grout properties. Grain size distribution and shape of lime particles and their aggregates in the water suspension are key parameters influencing the following fresh grout properties: fluidity, injectability, the mixture’s stability, and water retention capacity. However, the lime injection grouts’ mechanical strengths were higher in relation to an increase in the content of portlandite and the Blaine specific surface area of the dry hydrate.

## 1. Introduction

Hydrated lime was one of the prevailing binders for renders (external wall mortar layers), plasters (internal wall mortar layers) and masonry mortars up to the 20th century, when cement-based materials took the dominant role in the building sector. Unfortunately, cement-based materials were also applied to repair historic buildings where hydrated lime composites were used to bond the masonry units and protect masonry walls. Due to incompatibility with the historic masonry fabrics and additional unfavourable characteristics of the Portland cement binder—such as salt formation—the historic masonry buildings suffered new extensive damage. Over the last decades, the hydrated lime binder that provides similar composition and properties as the original historical architectural fabrics has become widely used to repair and restore historic lime plasters and renders. Where consolidation or re-attachment of such architectural surfaces is needed, architectural injection grouts prepared using the hydrated lime binder are often used to ensure compatibility between the new and historical materials and components [1]. A comprehensive state of the art regarding the composition of architectural injection grouts used in restoration practise between 1950 and 2015 is given in [2].

The reproducibility of the injection grouts’ properties is better when dry hydrated lime is applied as the binder. Moreover, the dry hydrate enables an easy application of different chemical admixtures in the grout mixtures—such as superplasticisers that reduce the water content of the grout considerably and thus increase its mechanical properties [3,4]. Additionally, a range of the lime-based grouts’ proportions are easily prescribed [5].

Historically, hydrated lime was produced by burning limestone in a lime kiln to obtain calcium oxide or quicklime, which was subsequently slaked with an excess of water in an exothermic reaction to form calcium hydroxide. Slaked lime or lime putty obtained in the process was stored in pits for at least three months before use. In the 20th century, industrial production fulfilled the need for ready-to-use hydrated lime in larger quantities, promoting hydrated lime as a dry powder [6]. In today’s industrial process, the production of dry hydrate is based on quicklime hydration with a controlled excess of water. Technically, the terms hydration and slaking are synonymous. That said, slaking involves a higher water amount and produces a wet hydrate in the form of lime putty, while hydration yields the dry powdered hydrate [7]. The dry hydrate is produced by mixing one part by weight of quicklime with about 0.5 to 0.75 parts of water. This value is significantly above the theoretical amount of water (0.245) required for complete hydration. A higher amount of water is necessary due to water evaporating during the hydration process [8].

The shape and particle size distribution of calcium hydroxide depend on the slaking process parameters. Several studies highlight the importance of the water/lime ratio, temperature and agitation rate on the final quality of lime putty and dry hydrate [7,9,10,11,12,13,14,15]. Whitman and Davis [11] studied the influence of different hydration processes on the properties of dry hydrated lime. Their study showed that a high-grade hydrate that contains many fine particles is produced when the rate of hydration is rapid compared to the particles’ growth rate. Excess water, reasonably high temperatures, and agitation all favour rapid hydration and a fine product. Another study [12] showed that a more reactive hydrate with a higher resistance to segregation in suspension is produced when the quicklime with finer particle size distribution is used. Dry hydration of quicklime should occur at around 100 °C to obtain a finer product [7,11,12].

The purity of quicklime is another parameter that influences calcium hydroxide quality. It depends on the quality of limestone and the manufacturing process. The main impurities consist of silica, alumina, iron and magnesium (in high calcium lime) [16]. Magnesium oxide (MgO) is disadvantageous in high calcium lime as it affects the reactivity of the quicklime [17]. Additionally, the four impurities listed influence limestone hydraulicity calculated using the cementation index (CI), developed by Eckel [18]. According to the CI value, the lime-based binders could be classified into five categories: pure (<0.15), subhydraulic (0.15–0.30), feebly hydraulic (0.3–0.5), moderately hydraulic (0.5–0.7), and eminently hydraulic (0.7–1.1) [19]. Impurities can also influence the optical properties of hydrated lime [9,16].

Many studies have compared the physical, chemical, and mechanical properties of lime powder and lime putty based mortars. Recent studies [6,20] show that mortars with dry hydrated lime have a higher carbonation rate and higher compressive and flexural strength values compared to the lime putty mortars subjected to ageing for more than 90 days. On the contrary, older studies promote the use of lime putty based mortars. Rodriguez-Navarro et al. [10] reported that the lime putty prepared with dry hydrate did not achieve traditional slaked lime putty’s properties, such as high workability, sand-carrying capacity, and good setting, strength development, and durability. The dry hydrated lime putty exhibited oriented aggregation of nanoparticles, which is irreversible and resulted in a significant decrease in specific surface area and, consequently, lower workability and slower reactivity. Elert et al. [7] also recommended using mortars prepared with the aged lime putty, which exhibit higher porosity and water-retention capacity. The porosity and water absorption of the dry hydrated lime mortars are greater than those of the lime putty mortars [6,21]. The plasticity of lime putties prepared using seven dry hydrated calcium limes was studied by Klein et al. [22]. The higher plasticity value is related to the higher specific surface area of dry hydrates due to the interaction between the liquid phase and the calcium hydroxide particles. A higher specific surface area is the result of the finer particle size distribution in hydrated lime. On the other hand, Paiva et al. [23] found that lime mortars prepared using dry hydrated lime and left to mature for a week, isolated from atmospheric CO_2_, present a thickening behaviour due to the agglomerates’ gradual breakdown and the increase in the surface area of particles exposed to the binding of water. The amount of free water was reduced, and the mortars became denser in their fresh state; they showed higher strengths at the age of 90 days and achieved a lower capillary absorption coefficient than the mortars that were not subjected to a maturation process. However, the authors concluded that increasing the rotation time and speed during the mixing process could achieve the same effect.

The use of dry hydrated lime and lime putty as a binder in architectural injection grouts is addressed mainly in combination with pozzolanic additives and/or chemical admixtures [2,24,25,26]. Relevant experimental studies [2,24,25,26,27,28,29] show that various key parameters can influence the properties of injection grout in its fresh and hardened state. These include grout composition, water-to-binder ratio, the reactivity of pozzolanic material used, chemical admixture type and content, incorporation of additives such as fibres and hollow glass bubbles, and the mixing and curing process. However, at the age of 90 days, the compressive strength of injection grouts based on dry hydrated lime [2,25] or lime putty [26], with or without pozzolanic constituent and/or superplasticiser, is in the range of 2.10 to 3.13 MPa.

The present study compares and evaluates the fresh and hardened properties of architectural injection grouts with the same composition. The grouts were prepared using three commercially available dry hydrated limes (powders) produced in Slovenia and Switzerland.

## 2. Materials and Methods

### 2.1. Materials and Composition

Three commercially available dry hydrated lime types were used to prepare the injection grouts. The limes are classified according to the EN 459-1:2015 standard [30]. The limes of classes CL 70-S (IAK, Kresnice, Slovenia) and CL 90-S (IGM, Zagorje, Slovenia) were produced in Slovenia (denotations SI-CL70 and SI-CL90) and the third, of class CL 90-S (KFN, Netstal, Switzerland), was produced in Switzerland (denotation CH-CL90). A finely ground limestone supplied from Slovenia (CALCIT, Stahovica, Slovenia) was used as a filler. The chemical compositions of the limes and limestone filler, determined by the X-ray fluorescence analysis (Bruker S8 TIGER, Anhovo, Slovenia) according to the EN 196-2:2013 standard [31], are shown in Table 1. Table 2 gives contents of crystalline phases in the three limes and filler, determined by the X-ray powder diffraction (XPert Pro X-ray diffractometer; measurement parameters: Cu-Kα radiation λ = 1.54 Å, exploration range from 20° and 70° 2θ, (University of Ljubljana, Ljubljana, Slovenia). The quantitative phase analysis of the samples was completed using the Rietveld method.

The results in Table 1 and Table 2 show that the CH-CL90 lime contains the highest portlandite content and a higher purity compared to the two limes from Slovenia. The highest content of impurities can be found in the SI-CL90 lime from Slovenia, which also contains the highest MgO content that can negatively influence the slaking process of the quicklime and thus the quality of the hydrated lime [17]. All these hydrated lime products can be characterised as high-calcium lime (Ca(OH)_2_ ≥ 90%) with traces of CaCO_3_ (≤6% by mass) [32].

The limestone filler is a very pure calcite powder, composed of 95.3% calcite and 4.7% dolomite (Table 2).

The specific surface area determined by the Blaine method [33] may be one of the hydrated limes’ most important physical properties [8]. The pycnometer method was applied to determine particle density [33]. Table 3 shows the Blaine specific surface area and density values for the studied dry hydrated limes and limestone filler. The SI-CL70, SI-CL90, and CH-CL90 limes have different fineness values; they are equal to 9623 cm^2^/g, 8767 cm^2^/g, and 16198 cm^2^/g, respectively. It is evident that the Slovenian limes SI-CL70 and SI-CL90 are much coarser than the CH-CL90 lime from Switzerland, which indicates that Slovenian limes possess lower reactivity. The particle density of a particular lime is well correlated (R^2^ = 0.997) to its specific surface area. The North America National Lime Association uses hydrated lime particle density to classify it as a high-calcium lime (densities between 2.3 and 2.4 g/cm^3^) [34]. According to this criterium, the CH-CL90 lime is the only one that can be classified as a high-calcium hydrate (particle density of 2.34 g/cm^3^). The particle densities of hydrated limes used in studies on lime-based mortars and grouts range from approximately 2.2 g/cm^3^ [35,36] to 2.47 g/cm^3^ [37]. The hydrated lime particles are characterised by their irregular block-like shape, with each particle being a porous cluster of small grains [38]. It can be concluded that a lower density of hydrated lime results from higher porosity of the cluster, where smaller particles present a reduced internal porosity.

The limestone filler particles had a density of 2.76 g/cm^3^ and water absorption of 0.4%; their maximum size was 100 µm, with 10%, 20%, 50%, and 90% of particles smaller or equal to 3 µm, 9 µm, 15 µm, and 40 µm, respectively.

The composition of grout mixtures in this study is based on the 1:3 (lime: filler) volume ratio composition from the previous study [2]. The volume ratios of the components in [2] were converted to mass ratios to provide identical compositions of the grouts tested. Each grout mixture was composed of 290 g lime, 1030 g filler, and 540 g water (water/binder ratio of 1.86). The grout’s adequate consistency and workability were assured using a polycarboxylate ether-based (PCE) super plasticiser. The super plasticiser content was equal to 0.5% of the total mass of solids (lime + filler).

The grout mixtures were prepared with a simple kitchen mixer to simulate the preparation of grout mixtures on a construction site. The mixer was a small hand-held electric whisk with a power of 300 W. The lime and filler were mixed first. This was followed by 70% of the water being added and mixed for 2 min at a low speed (540 rpm). In the last 15 s of the low-speed mixing, the PCE-SP and 30% of the water were added. Finally, each grout was mixed for 3 min at high speed (1200 rpm).

### 2.2. Methods

Test methods used to evaluate the properties of the grouts in the fresh and hardened state are predominantly the same as described in [28]. Thus, in continuation, a short list of the tests with essential information is given for procedures already described in [28], and a more detailed description is available for the rest of the tests. At least three repetitions of each test were carried out per grout mixture.

The fresh properties of the injection grouts were evaluated as follows:

The mini slump-flow test [39] was used to determine the flow behaviour of the grout mixtures.

According to the modified ASTM C940 standard procedure [40], the bleeding test was carried out. The modification applied reduced the grout volume from 800 ± 10 mL to 80 ± 1 mL.

The wet density of the grout was measured according to the modified EN 1015–6:1998 standard procedure [41]. The volume of the sample was reduced from 1000 mL to 10 mL [42].

The water-retaining ability of the grout was evaluated by the prEN 1015–8:1999 standard procedure [43].

The drying shrinkage test with mortar cups was used to determine the reduction in grout volume after hardening [42].

An injectability test with a syringe was used to determine the ability of the grout to fill a capillary network of dry granular materials under pressure [42]. First, 20 mL of the grout was poured into a vertically held syringe that was partially filled with 20 mL of granular material. Subsequently, the pressure was applied to the grout with a plunger. The crushed lime mortar was used as a granular material, with a grain size between 2 and 4 mm, which simulates an approximately 0.3–0.6 mm large crack or void width. After 10 min, the water absorption coefficient of the mortar was 11 kg/(m^2^√min). The injectability of the grout is classified as the following: easy (E)—if the grout flows through the granular material and out of the syringe tip when pressure is applied; feasible (F)—if the grout flows through the granular material and reaches the tip but does not flow through it; and difficult (DL)—if the grout stops in the granular material before reaching the tip [42]. The penetration distance, measured from the top of the granular material to the level of the grout, is recorded as L in millimetres.

The hardened properties of the gout samples were evaluated at the age of 90 days. The grouts were cast in cylindrical moulds, with a diameter and height equal to 50 mm, and demoulded after 48 h. Curing was executed under controlled ambient conditions (RH 60 ± 10% and 19 ± 1 °C) until the test day.

The grouts’ dry density and water absorption by capillarity were determined using the EN 1015-10:1999 [44] and EN 1015-18:2004 [45] standard procedures, respectively. The total and capillary porosities were measured in accordance with the Appendix A of Swiss standard SIA 262/ 1:2008 [46]. The compressive test was carried out in accordance with the EN 1015–11:1999/A1:2006 standard [47]. The splitting tensile test followed the ASTM C496/C496 M-1 standard [48]. The compressive and splitting tensile strengths were determined on four specimens per composition. Tests were performed by a Roel Amsler HA 100 servo-hydraulic testing machine (Zwick GmbH & Co. KG, Ulm, Germany), complemented by a load cell with the capacity adjusted to the compressive (25 kN) and splitting tensile (5 kN) strength of the tested specimens.

## 3. Results and Discussion

### 3.1. Fresh State Properties

The average values and the corresponding standard deviations of wet density, mini-slump flow, and bleeding after 3 h, and water retention capacity of the SI-70, SI-90, and CH-90 grouts are listed in Table 4. The fresh state properties of the SI-70 and CH-90 grouts are approximately the same. The SI-90 grout, on the other hand, has a higher mini-slump-flow value and increased bleeding compared to the SI-70 and CH-90 grouts. As all constituents in the studied mixtures were of the same mass, the hydrated lime particle density (Table 3) and entrapped air content—as a result of mixing and casting procedures—determined the actual volumes of the lime particles, water, and limestone filler in the grout’s unit volume. This is reflected in the fresh grouts’ densities. The mini-slump-flow value, which is a measure of flowability and consistency of the fresh grout, is often related to the paste’s yield stress τ_0_. “Paste” is a generic term for the mixture of binder, water and filler particles that are smaller than 0.1 mm; it can also contain a chemical admixture. The highest mini slump-flow value of 300 mm was measured for the SI-90 mixture, prepared with the coarsest lime. The two finer limes had a 15% lower slump-flow value compared to that of the SI-90 mixture.

Ince et al. [49] observed a loss of mortars’ fluidity due to the finer particle size distribution of the hydrated lime. By maintaining the standard Vicat consistency of lime putties, Klein et al. [22] showed that the particle size distribution of dry hydrated lime affected the plasticity of the lime putty, which increased with decreasing lime particles’ size. Plasticity is a rheological property that relates to lime putty workability. Both approaches confirmed the vital influence of dry hydrated lime fineness on the fluidity and workability of the fresh lime mortars and grouts. It can be concluded that dry hydrated limes with higher total specific surface areas of particles and aggregates require higher mixing water content to produce a water suspension (hydrated lime putty) with the same consistency. This is related to the water physically adsorbed onto the surface of solids in suspension. When using the mini-slump-flow test method to evaluate the flowability and consistency of lime grout, the relation between water content and relative flow area (R = (slump–flow value/100)^2^ − 1) is often considered as a parameter that measures the sensitivity of the grout flowability to increasing water content [39]. The retained water, which provides sufficient particle dispersal for flow to commence, is comprised of the water adsorbed onto solid particles and that which is required to fill the voids in the powder system. A water content higher than the retained water content is needed for a slump–flow value of more than 100 mm. At the constant water content in the grout mixtures, lime particles with a higher surface area physically bind more water, and thus less water is available for the grout to flow. The oriented aggregation of dry hydrated lime particles in the fresh grout could be the reason for the surface area reduction and the change in a grout’s workability [10]. These observations explain the similar flowability of the SI-70 and CH-90 grouts, which could result from the finest CH-CL90 lime particles’ oriented aggregation in the grout suspension.

**Figure 1:**
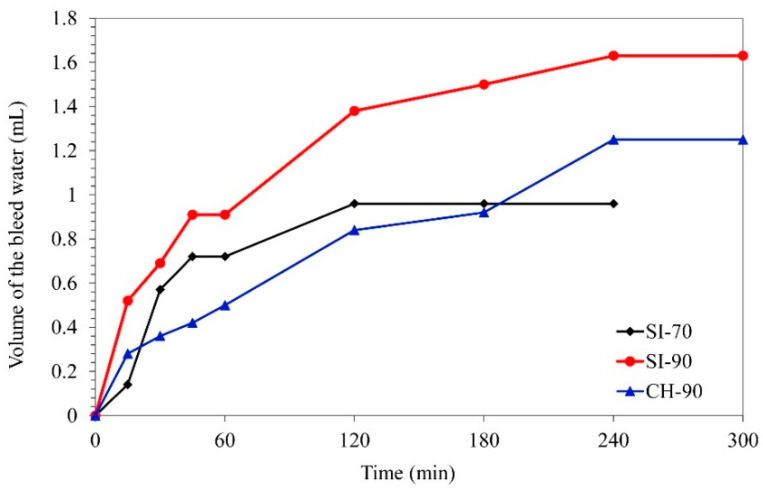
The bleed water volume, measured over five hours for the three grout compositions.

Figure 1 shows the highest volume of bleed water accumulating on a particular fresh grout surface at prescribed intervals. After 3 h of testing, the grout mixture SI-90 exhibited a higher average bleeding (1.5%) than mixtures SI-70 and CH-90, where the average bleeding was 1.0 and 0.9%, respectively (Table 4). However, the final average bleeding—measured after 5 h, when the two successive measurements showed no further bleeding—was increased to 1.6% for the SI-90 grout and 1.3% for the CH-90 grout. On the other hand, the mixture SI-70 showed no further changes in bleed water up to the 5th hour of testing (Figure 1). The results of the bleeding test indicate a faster segregation and a subsequently reduced stability of the coarser dry hydrate and a much slower bleeding of the finest dry hydrate (Figure 1). However, the final bleeding appears not to be directly related to the measured Blaine specific surface area of the dry hydrated lime. Again, a possible oriented aggregation of lime particles in the water suspension [10] can explain the observed bleeding behaviour of the CH-90 grout. The smaller Ca(OH)_2_ particles and their aggregates provide a larger surface area for wetting and bonding with the filler particles, which improves the stability of the mixture and, consequently, its resistance to bleeding [50]. Moreover, additional influencing parameters—such as the packing of particles, oriented Ca(OH)_2_ aggregates, impurities, etc.—can impact the stability of the grout [7,10]. Oriented aggregates reduce the packing density of solid particles in the suspension and thus increase the retained water content. Increased volume of the retained water filling voids between the particles can be responsible for the time-dependent bleeding observed in the CH-90 grout.

Although there is no significant difference in the water retention capacity of the studied grouts (Table 4), due to the test method’s poor repeatability, the test results indicated a lower water retention capacity for the coarser SI-CL90 lime. The highest average water retention capacity of 85% was measured for the CH-90 grout and the lowest (82%) for the SI-90 grout. These results are in line with the results of previous studies [49,51], where it was shown that the water retention capacity increases with a decrease in the particle size of the lime. Ince et al. [50] attributed the observed behaviour to the small radii of curvature of the menisci between finer particles, which could contribute to the increased water-retaining ability of the grout. Biçer-Simsir et al. [42] pointed out that the high water retention capacity of the grout is an essential property for reducing the grout’s shrinkage and achieving satisfactory values for other properties.

Table 5 presents the results of the injectability test for the dry and pre-wetted crushed lime mortar used as granular material inside the syringe. The SI-70 and SI-90 grouts’ injectability was classified as easy (E) through dry and pre-wetted granular material. The situation was significantly different for the CH-90 mixture, which only penetrated 25 mm of the dry granulate and was thus classified as difficult (D_25_). When the granulate was pre-wetted, the injectability of the CH-90 improved considerably and was classified as feasible.

The injectability of the grout is closely related to its fluidity, viscosity, and water retention ability. The mixture SI-90, with the highest fluidity and water release, achieved a level of injectability comparable to that of the SI-70 mixture, which exhibited a lower fluidity and similar water retention. The poorest injectability was observed in the CH-90 grout. Oriented Ca(OH)_2_ aggregates that could form in the CH-CL90 grout result in higher resistance of the grout to the applied pressure and, consequently, in a loss of injectability of the CH-90 grout. This grout would thus require an addition of water to its composition to reach adequate fresh state properties. 

**Figure 2:**
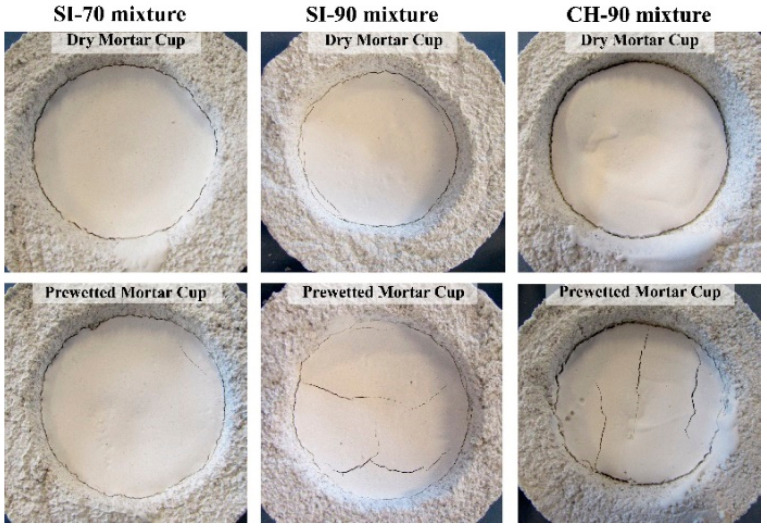
Grout mixtures SI and CH after drying in mortar cups.

The results of the drying shrinkage test inside mortar cups are presented in Table 6. Figure 2 shows the volume change for the mixtures SI-70, SI-90 and CH-90 after drying in the dry or pre-wetted mortar cups. The SI-70 and SI-90 grouts formed an approximately 0.2 mm thick separation ring (a continuous circular gap between the cup and the grout in Figure 2) between the grout and mortar cup in the dry and prewetted cups. A much thicker separation ring of 0.4 mm formed in the CH-90 grout, again in dry and pre-wetted mortar cups. The SI-90 mixture also formed cracks in the grout—small cracks with a maximum width of 0.1 mm close to the cup’s wall (dry mortar cups) and extensive cracks of 0.2 mm width (pre-wetted mortar cups). The mixtures SI-70 and CH-90 did not form cracks in the grout when dry mortar cups were used. On the other hand, extensive cracks of 0.3 mm were formed in the CH-90 grout’s pre-wetted cups.

The mortar cup shrinkage test simulates the absorption effect of porous mortar and demonstrates the grout’s ability to retain moisture inside its structure when exposed to dry for an extended period (24 h or more). The obtained test results indicate that the SI-70 grout possesses the best resistance to the suction of porous mortar and air drying and thus the best volume stability. The lowest volume stability was obtained for the CH-90 grout with relatively high final bleeding and the lowest injectability/penetrability, resulting in a lower bond strength between the grout and the mortar cup. The SI-90 grout’s volume stability was only slightly better than that of the CH-90 grout.

Carbonation shrinkage is another mechanism that influences the hydrated lime grout shrinkage. As the mechanism is basically the same as in the case of drying shrinkage, it is often considered part of the drying shrinkage. Swenson and Sereda [52] suggested that carbonation shrinkage occurs due to a gradient of moisture content within the CaCO_3_ passivation layer around noncarbonated portlandite; it is the highest when the relative humidity of the air is approximately 50%. They also indicated that the lime binder’s rate and degree of portlandite carbonation and carbonation shrinkage increase with the lime fineness [52]. These findings explain the highest shrinkage deformations and cracking observed for the CH-90 lime grout.

From the fresh properties’ point of view, the SI-70 grout showed the best overall performance appropriate for the practical application.

### 3.2. Properties in Hardened State

Table 7 reports the results of dry density, total and capillary porosity, and water absorption coefficient after 24 h and 10 min for the SI-70, SI-90, and CH-90 hardened grouts at the age of 90 days. The average dry density of the grouts ranged from 1.45 to 1.51 g/cm^3^. Higher values measured for the SI-70 and CH-90 grouts (1.50 g/cm^3^ and 1.51 g/cm^3^) can be predominantly related to the carbonation effect due to a higher portlandite (Ca(OH)_2_) content (Table 2). They can also be linked to the faster reactivity of smaller lime particles that form more calcite (CaCO_3_) in 90 days compared to the coarser SI-CL90 lime with the lowest portlandite content, as explained in [6,52].

The total porosity results show that the denser SI-70 and CH-90 mixtures have a higher total porosity (43–44%) compared to the SI-90 mixture which exhibits the lowest dry density—predominantly due to a higher entrapped air content (6%) inside the specimens. However, all three mixtures showed approximately the same capillary porosity (37–38%), much smaller than the initial volume of water in the grouts (about 52%). The water absorption of the limestone filler (0.4%) resulted in only 4 g of water absorbed by the filler for all three compositions. Therefore, it did not have an important influence on the capillary porosity formed due to evaporation of the excess of kneading water from residual spaces previously occupied [53]. Due to capillary forces, the evaporation of water results in the shrinkage of lime grout and thus in about 3 to 5% lower initial porosity of the dry noncarbonated grout compared to the volume percentage of kneading water [54]. Moreover, the mercury intrusion porosimetry of fully carbonated lime showed an open porosity that is about 10 to 12% lower than that of the noncarbonated grout [36]. These findings are in line with results in Table 7, where capillary porosity of about 38% is 14% lower than the volume percentage of kneading water in the lime grouts (52%).

**Figure 3:**
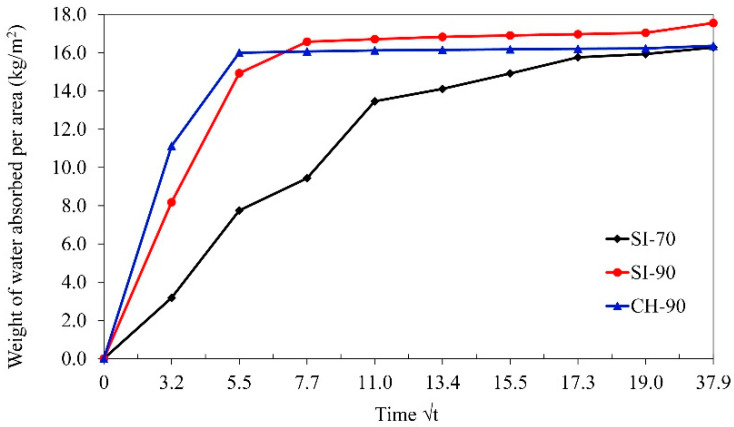
Capillary water absorption curves of the three injection grouts.

From Table 7 and Figure 3, it can be seen that all three grouts absorbed about the same water content after 24 h, which is consistent with their capillary porosities. However, there is an important difference in the initial capillary sorptivity of the grouts, determined after 10 min; the mixture CH-90 absorbed water much faster (10.6 kg/m^2^) than the SI-90 mixture (8.2 kg/m^2^). Considerably slower initial sorptivity of 3.2 kg/m^2^ was obtained for the SI-70 grout. As the capillary sorptivity force (as a pressure difference) increases when the pore diameter drops [55], we can conclude that the most refined capillary pore system was formed in the CH-90 grout and the coarsest in the SI-70 grout.

**Figure 4:**
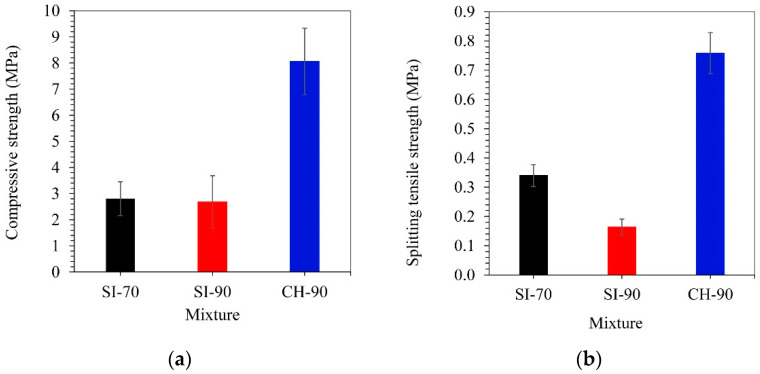
(**a**) Compressive and (**b**) splitting tensile strength for SI and CH mixtures.

**Figure 5:**
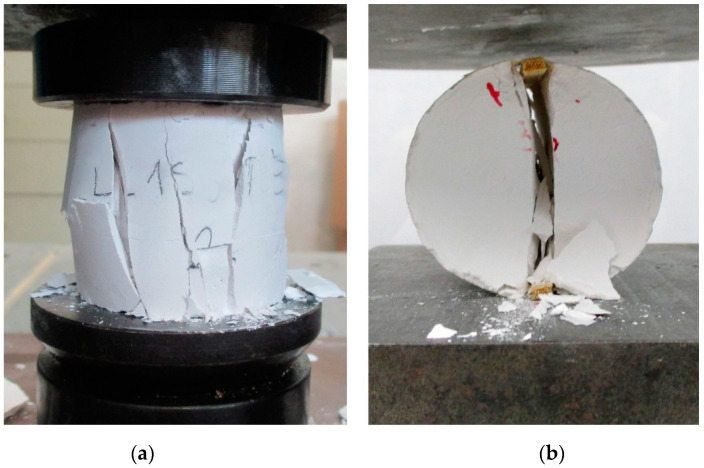
Examples of failure modes: (**a**) Compressive strength and (**b**) splitting tensile strength for SI and CH mixtures.

Figure 4 shows the compressive and splitting tensile strengths of the lime grouts at 90 days. The failure modes for compressive and splitting tensile strengths are shown in Figure 5. The average compressive and tensile strengths for the SI-70 and SI-90 grouts are in the range expected for materials with a pure hydrated lime binder—approximately 2.8 MPa and 0.16–0.34 MPa, respectively. The compressive strengths of hydrated lime-based mortars and injection grouts range from 0.2 to 4.5 MPa, with tensile strengths of between 0.07 and 1.5 MPa [2,6,21,51,56,57]. On the other hand, the CH-90 grout reached unexpectedly high compressive and tensile strengths equal to 8.1 and 0.76 MPa, respectively. These values are about 3 times higher than those seen in grouts prepared with Slovenian limes. The CH-90 grout compressive strength lies within the interval of 1.0 to 14.0 MPa, which represents the compressive strengths reported for natural hydraulic lime or lime-based mortars with pozzolanic additives [2,25,26,51,56,58,59]. Moreover, the combination of hydrated lime CH-CL90 and PCE superplasticiser leads to compressive and tensile strengths close to the upper limits of the intervals 3–8 MPa and 0.3–1.2 MPa, respectively. These intervals were reported by Ferragni et al. [60] for hydraulic lime architectural injection grouts.

Dry hydrated lime properties that govern the grout strengths include impurities (Table 1), portlandite content (Table 2), and specific surface area of the lime particles. The smaller particles are more reactive compared to coarser particles (Table 3). The CH-CL90 lime is very pure, with the highest portlandite content and the highest specific surface area. Therefore, its carbonation rate and final volume of CaCO_3_ in the grout should be the highest among studied limes [52]. This conclusion is also in line with nanoscience findings; application of so-called “nano-lime” showed that reducing the size of calcium hydroxide particles leads to higher suspension stability. Moreover, smaller particles have a higher reactivity to form a calcite crystal structure, which improves the cohesion and mechanical strength of the wall paintings’ substrate layers [61]. Incorporating the PCE super plasticiser into the grout composition, combined with high-speed mixing (1200 rpm), disintegrates the lime and limestone filler particles agglomerates in the suspension, thus increasing the surface available for carbonation in the hardened grout. Simultaneously, a more homogeneous and compact hardened grout with much finer capillary pores is formed in the case of the CH-90 grout (Table 7). Fernandez et al. [62] and Silva et al. [63] showed that PCE superplasticiser in the lime mortar results in a strong reduction in macropores (in the diameter range of 1–10 µm) and a more compact, homogeneous and continuous mortar matrix. Compared to the lime mortar with the same water-to-binder ratio and without the PCE super plasticiser, compressive and flexural strengths increase considerably, especially at the mortar’s age of 6 months and one year. However, the porosity reduction can obstruct an adequate CO_2_ flow through lime-based mortars and grouts. Therefore, an open homogeneous microstructure supports a better and efficient carbonation rate and promotes a well-developed carbonate morphology [64,65]. Padovnik et al. [2] showed that the PCE superplasticiser was very effective at lowering the amount of water in hydrated lime based grout, which increased its mechanical strengths. According to Van Balen [66], one of the mechanisms that control the carbonation rate can be the dissolution of portlandite at the water-adsorbed surface; therefore, the carbonation rate depends on the hydrated lime specific surface area. Paiva et al. [23] stated that intense, rapid mixing might break up the hydrated lime particle aggregates, causing a reduction in the capillary water absorption and a mechanical strength increase.

In addition to the beneficial properties of the CH-CL90 lime in relation to strength properties of the architectural grout (as discussed earlier), there must be other influencing parameters responsible for such high compressive and tensile strengths of the CH-90 grout. The load-bearing capacity of the crystal lattice, which makes up the solid mass of the hardened grout, must be significantly higher for the CH-90 grout than the SI-70 and SI-90 grouts. It appears that the CH-CL90 lime (Table 3) can bind limestone filler particles more efficiently compared to the Slovenian limes and can form a composite solid mass with highly increased strength. The absence or reduced content of pre-existing cracks due to shrinkage, combined with the refined capillary porosity, could present another parameter responsible for the high strengths observed. In [67], authors showed that pre-existing cracks and their inclination angles to nearby entrapped air pores influence the loadbearing behaviour of the mortar.

## 4. Conclusions

This study addresses the influence of three dry hydrated limes on architectural injection grouts’ fresh and hardened properties. The main differences between the hydrated limes could be found in their chemical and mineralogical compositions, particle density, and specific surface area values, which indicate that the industrial production and limestone composition were not the same for the studied limes. The grouts’ composition was identical regarding mass ratios between lime, water, limestone filler and PCE super plasticiser. The PCE super plasticiser, combined with high-speed grout mixing (1200 rpm), disintegrates the lime and limestone filler particle agglomerates in the suspension and thus influences the fresh grout properties and the increased surface available for carbonation in the hardened grout. Additionally, oriented Ca(OH)_2_ aggregates can gradually form in the fresh grout and change the fresh grout properties. The main conclusions of this study can be summarised as follows.

The mixtures’ consistency, stability, water-retaining ability, and injectability were influenced by the specific surface area of the lime binder in the grout, governed by its particle and agglomerate size, distribution, and shape. Although the highest Bleine specific surface area was measured for the dry hydrated CH-CL90 lime, the CH-90 grout’s fresh properties were approximately the same compared to the SI-70 grout prepared with lower Blaine specific surface area dry hydrate. The only exception was injectability, which was the poorest for the CH-90 grout. Possible oriented aggregation of the CH-90 lime particles in water suspension can explain the observed behaviour. The SI-70 grout showed the most balanced fresh properties among the three grouts, most probably due to the appropriate combination of lime particles specific surface area and solid particles (lime and limestone filler particles) packing property.

The shrinkage test in mortar cups revealed an important influence of carbonation shrinkage on the CH-90 grout. The rate and degree of portlandite carbonation were the highest among the studied grouts, which resulted in extensive carbonation shrinkage. As a consequence, poor volume stability of the CH-90 grout was observed in the study.

The three grouts showed the same capillary porosity, which is consistent with the same water content of the fresh mixtures and the same water content absorbed in the hardened state. On the other hand, faster carbonation and a higher volume of CaCO_3_ in the CH-CL90 lime did not reflect in the CH-90 grout open porosity. There was an important difference in the initial capillary sorptivity of the grouts (determined after 10 min) that indicated the formation of the most refined capillary pore system in the CH-90 grout, while the coarsest capillary pore system appeared in the SI-70 grout.

The CH-CL90 lime resulted in an unexpectedly high compressive and tensile strength of the CH-90 grout at 90 days, 8.1 and 0.76 MPa, respectively. These strengths are about 3-times the strengths measured for the SI-70 and SI-90 grouts. They are close to the upper limit of the intervals reported by Ferragni et al. [37] for hydraulic lime architectural injection grouts. The main parameters that increase the lime grout strengths are the highest portlandite content and specific surface area of the lime particles and more homogeneous and compact hardened grout with the finer capillary network that still provides efficient carbonation. Additionally, the much higher particle density of the CH-CL90 lime and its ability to bind limestone filler particles efficiently can form a composite solid mass with highly increased load-bearing ability. Further work is required to evaluate the microstructure of limes and grout mixtures and provide answers to the remaining questions.

The use of dry hydrated limes is increasingly recommended, as they have a lower mass volume and facilitate a range of mix proportions and reproducibility of the properties in a fresh and hardened state. The application of the PCE super plasticiser can significantly reduce the kneading water in the dry hydrated lime-based mixtures, which, in turn, compensates for the deficiencies compared to the hydrated lime putty mixtures. Moreover, in the case of architectural injection grouts, the quantities of dry hydrated lime are low enough to use the best possible product available on the international market. In Slovenia, for example, products available in other European countries should be reviewed. The Swiss product used in this study showed that commercially available dry hydrated limes could possess highly diverse properties and provide high enough strengths for different applications, especially in historical structures’ repair and strengthening.

## Figures and Tables

**Table 1:** Chemical composition of the dry hydrated limes and limestone filler.

Sample	CaO (%)	MgO (%)	Al_2_O_3_ (%)	Fe_2_O_3_ (%)	SO_3_ (%)	SiO_2_ (%)	I.L. (%)
SI-CL70 lime	71.25	2.09	0.60	0.19	0.06	0.79	25.69
SI-CL90 lime	71.01	3.05	0.58	0.20	0.14	2.14	23.38
CH-CL90 lime	74.90	0.40	0.02	0.01	0.02	0.05	25.00
Limestone filler	55.38	0.76	0.15	0.01	0.01	<0.01	44.02

**Table 2:** Contents of crystalline phases in the powders, obtained by the Rietveld method.

Sample	Portlandite (Ca(OH)_2_)	Calcite (CaCO_3_)	Periclase (MgO)	Quartz (SiO_2_)	Lime (CaO)	Magnesite MgCO_4_	Larnite (Ca_2_SiO_4_)	Dolomite (CaMg(CO_3_)_2_)
SI-CL70 lime	95.8	2.9	0.2			0.3	0.8	
SI-CL90 lime	92.5	1.2	2.3	0.2	0.1	0.4	3.5	
CH-CL90 lime	97.0	3.0						
Limestone filler		95.3						4.7

**Table 3:** Particle density and specific surface area of hydrated limes and limestone filler.

Sample	Particle Density (g/cm^3^)	Blaine Fineness (cm^2^/g)
SI-CL70 lime	2.237	9623
SI-CL90 lime	2.217	8767
CH-CL90 lime	2.343	16,198
Limestone filler	2.764	3194

**Table 4:** Wet density (g/cm^3^); mini-slump flow (mm); bleeding after 3 h (%); and water retention capacity (%) of the fresh SI and CH mixtures.

Mixture	Wet Density (g/cm^3^)	Mini Slump Flow (mm)	Bleeding after 3 h (%)	Water Retention Capacity (%)
SI-70	1.74 ± 0.02	259 ± 16	1.0 ± 0.3	83 ± 1
SI-90	1.74 ± 0.00	300 ± 15	1.5 ± 0.3	82 ± 2
CH-90	1.76 ± 0.02	254 ± 10	0.9 ± 0.4	85 ± 2

**Table 5:** Results of injectability with a syringe.

Mixture	Crushed Lime Mortar	
	Dry	Wet
SI-70	E	E
SI-90	E	E
CH-90	D_25_	F

**Table 6:** Drying shrinkage in dry and pre-wetted mortar cups.

Mixture	Dry Mortar Cup		Pre-Wetted Mortar Cup	
	Separation Size (mm)	Crack Size (mm)	Separation Size (mm)	Crack Size (mm)
SI-70	0.2	0	0.2	0.1
SI-90	0.2	0.1	0.2	0.2
CH-90	0.4	0	0.4	0.3

**Table 7:** Density of hardened state (g/cm^3^); total and capillary porosity (%); and water absorption coefficient after 24 h (W_24_) and 10 min (W_10_) of the SI and CH mixtures.

Mixture	Dry Density (g/cm^3^)	Total Porosity (%)	Capillary Porosity (%)	Content of Air Pores (%)	W24 (kg/(m^2^√min))	W10 (kg/(m^2^√min))
SI-70	1.51 ± 0.01	43 ± 0	37 ± 0	6	0.43 ± 0.02	1.01
SI-90	1.45 ± 0.01	40 ± 1	38 ± 1	2	0.46 ± 0.01	2.59
CH-90	1.50 ± 0.02	44 ± 0	38 ± 0	6	0.43 ± 0.03	3.34

## Data Availability

Data sharing is not applicable to this article.
